# Autologous glioma cell vaccine admixed with interleukin-4 gene transfected fibroblasts in the treatment of patients with malignant gliomas

**DOI:** 10.1186/1479-5876-5-67

**Published:** 2007-12-19

**Authors:** Hideho Okada, Frank S Lieberman, Kevin A Walter, L Dade Lunsford, Douglas S Kondziolka, Ghassan K Bejjani, Ronald L Hamilton, Alejandro Torres-Trejo, Pawel Kalinski, Quan Cai, Jennifer L Mabold, Howard D Edington, Lisa H Butterfield, Theresa L Whiteside, Douglas M Potter, S Clifford Schold, Ian F Pollack

**Affiliations:** 1 Department of Neurological Surgery, University of Pittsburgh School of Medicine, Pittsburgh, PA, USA; 2 Department of Surgery, University of Pittsburgh School of Medicine, Pittsburgh, PA, USA; 3 Department of Neurology, University of Pittsburgh School of Medicine, Pittsburgh, PA, USA; 4 Department of Immunology, University of Pittsburgh School of Medicine, Pittsburgh, PA, USA; 5 Department of Medicine, University of Pittsburgh School of Medicine, Pittsburgh, PA, USA; 6 Department of Pathology, University of Pittsburgh School of Medicine, Pittsburgh, PA, USA; 7 Biostatistics Department, University of Pittsburgh Graduate School of Public Health, Pittsburgh, PA, USA; 8 Brain Tumor Program, University of Pittsburgh Cancer Institute (UPCI), Pittsburgh, PA, USA

## Abstract

**Background:**

The prognosis for malignant gliomas remains dismal. We addressed the safety, feasibility and preliminary clinical activity of the vaccinations using autologous glioma cells and interleukin (IL)-4 gene transfected fibroblasts.

**Methods:**

In University of Pittsburgh Cancer Institute (UPCI) protocol 95-033, adult participants with recurrent glioblastoma multiforme (GBM) or anaplastic astrocytoma (AA) received gross total resection (GTR) of the recurrent tumors, followed by two vaccinations with autologous fibroblasts retrovirally transfected with TFG-IL4-Neo-TK vector admixed with irradiated autologous glioma cells. In UPCI 99-111, adult participants with newly diagnosed GBM or AA, following GTR and radiation therapy, received two intradermal vaccinations with the TFG-IL4-Neo-TK-transfected fibroblasts admixed with type-1 dendritic cells (DC) loaded with autologous tumor lysate. The participants were evaluated for occurrence of adverse events, immune response, and clinical response by radiological imaging.

**Results and Discussion:**

In UPCI 95-033, only 2 of 6 participants received the vaccinations. Four other participants were withdrawn from the trial because of tumor progression prior to production of the cellular vaccine. However, both participants who received two vaccinations demonstrated encouraging immunological and clinical responses. Biopsies from the local vaccine sites from one participant displayed IL-4 dose-dependent infiltration of CD4^+ ^as well as CD8^+ ^T cells. Interferon (IFN)-γ Enzyme-Linked Immuno-SPOT (ELISPOT) assay in another human leukocyte antigen (HLA)-A2^+ ^participant demonstrated systemic T-cell responses against an HLA-A2-restricted glioma-associated antigen (GAA) epitope EphA2_883–891_. Moreover, both participants demonstrated clinical and radiological improvement with no evidence of allergic encephalitis, although both participants eventually succumbed with the tumor recurrence. In 99-111, 5 of 6 enrolled participants received scheduled vaccinations with no incidence of major adverse events. Monocyte-derived DCs produced high levels of IL-12 p70. Treatment was well tolerated; however, we were unable to observe detectable IFN-γ post-vaccine responses or prolonged progression-free survival in these participants.

**Conclusion:**

Feasibility challenges inherent in the generation of a patient-specific gene transfection-based vaccine strongly suggests the need for more practical formulations that would allow for the timely administration of vaccines. Nevertheless, successful generation of type-1 DCs and preliminary safety in the current study provide a strong rationale for further efforts to develop novel glioma vaccines.

## Background

Although cellular mechanisms underlying the "immunologically privileged" status of the brain and brain tumors have been increasingly well-characterized during the past decade [1], it has also become apparent that this privileged status is not absolute. Immunotherapy may develop as an effective and safe treatment modality for malignant gliomas, a disease that continues to have a poor prognosis [reviewed in [2]].

Previous studies indicate that active immunization with tumor cells genetically engineered to express cytokines may positively influence the outcome of an anti-tumor immune response [3,4], and this hypothesis is supported by clinical responses of patients with melanoma [5].

With regard to vaccine strategies against CNS gliomas, we have compared various cytokines and delivery modes to find the most efficacious strategy of cytokine gene therapy against intracranial (IC) tumors in the rat 9L gliosarcoma model [6,7]. In these preclinical studies, we observed that peripheral immunization with IL-4 transfected rat 9L cells achieved the most potent therapeutic benefit [7]. To gain additional mechanistic insight into the efficacy of this approach, we evaluated the effects of transgene-derived IL-4 on endogenous cytokines, such as IL-12 and interferon (IFN)-γ [8]. These type-1 cytokines were necessary for long-lasting protective memory response against the tumor, and local paracrine production of IL-4 in vaccine sites promoted the accumulation and maturation of IL-12-secreting tumor-infiltrating DCs, which were capable of inducing tumor-specific CTL responses. These results suggest that vaccines consisting of tumor cells engineered to produce the type-2 cytokine IL-4 critically depend on type-1 immunity for their observed therapeutic efficacy. Indeed, IL-4 plays an important role in DC maturation [9] and promotes IL-12 secretion from DCs [10-12].

With regard to the vector design to deliver IL-4, to enhance the safety of gene-transfected vaccines, we incorporated the *Herpes Simplex Virus-Thymidine Kinase *(HSV-TK) downstream of IL-4 in our retroviral vector construct (TFG-hIL4-Neo-TK) [13]. Vaccination was effective in animals that received gancyclovir (GCV) treatment to eliminate live tumor cells persisting after the local host immune response to the vaccine [6].

On the basis of these observations, we developed a phase I clinical trial of vaccination with autologous tumor cells and autologous fibroblasts that are transfected with the TFG-hIL4-Neo-TK to evaluate feasibility, safety, local and systemic immunological response, as well as preliminary therapeutic activity of the approach in patients with recurrent malignant glioma (UPCI95-033) [14]. As IL-4 promotes maturation and IL-12 production of DCs, we subsequently developed another trial for patients with newly diagnosed malignant glioma, using TFG-hIL4-Neo-TK-transfected fibroblasts and type-1-promoting DCs [15] loaded with autologous glioma lysate (UPCI 99-111) [16]. In both trials, generation of sufficient gene-transfected fibroblasts required at least 7 to 8 weeks, which posed a major feasibility issue, especially in patients with recurrent malignant glioma. Nevertheless, successful generation of type-1 DCs and preliminary safety in the current study provide a strong rationale for further efforts to develop novel glioma vaccines.

## Methods

### Patients

Eligible patients were at least 18 years of age with histologically confirmed primary (UPCI 99-111) or recurrent (UPCI 95-033) supratentorial GBM or AA. Other eligibility criteria included the following: 1) Tumors had to be suitably located for subtotal resection; 2) resected tumor cells must be viable and sterile; 3) Karnofsky performance status ≥ 60% prior to surgery and prior to receiving the vaccine; 4) no evidence of systemic infection; 5) negative HIV test and negative hepatitis B surface antigen test prior to the first vaccination; 6) patients had to be off corticosteroids for at least two weeks prior to, and during the vaccine treatment; and 7) adequate liver, hematopoetic and renal function (WBC > 2000 cells/mm3, platelet > 50,000 cells/mm3, bilirubin < 2 mg/dl, creatinine < 2 mg/dl). Written informed consent was obtained for publication from the patients and/or their relatives.

### Obtaining autologous glioma cells and fibroblasts for vaccines

Methods and procedures for processing autologous glioma cells and fibroblasts were detailed previously [14,16,17]. In brief, for glioma cells, the UPCI Tissue Bank obtained surgical specimens directly from the operating room after a surgical pathologist had reviewed the tissue to ensure adequate diagnostic material had been obtained, and released the remainder to the Tissue Bank personnel. Tumor cells were recovered from Ficoll-Hypaque gradients and washed in tissue culture medium. Dissociation of glioma tissues was conducted under sterile conditions in a laminar flow hood by a certified Tissue Bank technician. After mechanical dissociation and washing, tissue fragments were digested with 0.14% collagenase Type I (200 units per mg; Sigma C/0130) and 0.1% DNAse Type I (500 Kunitz units per ml; Sigma D/0876) at 37°C for 30 to 45 minutes. Then, free cells were decanted through three layers of sterile medium wet nylon mesh (163T; Martin Supply Company, Baltimore, MD) into a 50 ml centrifuge tube. After washing, cells were pooled and counted, and the cell viability assessed by the Trypan blue exclusion test.

Skin harvest for establishing fibroblast cultures was obtained from each patient by either excision of a portion of the skin (approximately 3 cm by 6 cm) from the torso at time of craniotomy (95-033) or punch-excision (7 mm in diameter) from the torso under local anesthesia (99-111). The skin grafts were minced using a surgical scalpel into 1–2 mm^2 ^pieces, which were then cultured in T25 plastic culture flasks with culture medium supplemented with 10% human AB serum, 5 μg/ml basic fibroblast growth factor and antibiotics, and were allowed to attach to the plastic (30–60 min) at 37°C in an atmosphere of 5% C0_2 _in air. Then, the flasks were incubated undisturbed for 5 to 7 days. When the fibroblasts had expanded sufficiently, they were briefly (up to 10 min) incubated with 0.25% trypsin in EDTA (Gibco, culture grade) at 37°C, and then detached cells were washed and transferred to a fresh flask in culture medium.

### Transfection and growth of fibroblasts

Retroviral vector supernatant from a ψCRIP/TFG-hIL4-neo-TK producer cell line master cell bank was prepared in the Human Gene Therapy Applications Laboratory (HGTAL) within the University of Pittsburgh [13]. The safety criteria requirements of the supernatant and master cell bank have been previously reported [14,16]. Although we had planned, if possible, to transfect patient-derived glioma cells in UPCI 95-033, these cells were not successfully transfected with the clinical grade vector supernatant. Therefore, we used fibroblasts transfected with TFG-hIL-4-neo-TK admixed with irradiated glioma cells in UPCI 95-033. UPCI 99-111 was designed to use autologous fibroblasts transfected with TFG-hIL-4-neo-TK, which were admixed with tumor lysate-loaded DCs for the final vaccine formulation. Cultured fibroblasts were transfected as soon as significant growth was observed according to our previously described methods [13,14,16]. Twenty-four hours after transfection, these cells were then exposed to the selective pressure of 0.3 mg/ml of G418 to facilitate isolation of transfected cells.

### Generation of lysate-loaded DCs

For generation of type-1 DCs in UPCI 99-111, participants' whole blood was obtained by conventional peripheral blood draw (up to 100 ml/draw). Peripheral blood mononuclear cells (PBMC) were isolated by Ficoll-Paque PLUS (Pharmacia, Piscataway. NJ), and were plated onto plastic flasks (10–15 × 10^6 ^PBMC/T-25 flask) and incubated in 37°C and 5% CO_2 _for 45 minutes. Plastic-adherent monocytes were cultured in Iscove's-modified Dulbecco's medium containing 10% fetal bovine serum (Certified US origin, Gibco), 250 IU/ml recombinant human (rh) IL-4 and 500 IU/ml rh granulocyte macrophage-colony stimulating factor (GM-CSF) (FDA-approved reagents) for 6 days. Cryopreserved glioma cells were thawed, and then lysed by 5 cycles of freeze and thaw in PBS. After centrifugation, the lysate was obtained as the supernatant, then added to the DC culture, together with the mixture of maturation-inducing and type-1 polarizing cytokines (1000 U/ml IFN-γ, 20 ng/ml IL-1β and 50 ng/ml TNF-α). One T-25 flask of cells not given this cytokine mixture served as a control in assessing the IL-12 production capability of type-1 polarized vs. standard DCs. At 16 hours after the addition of tumor lysate, the cells were washed and prepared for vaccination. On the vaccination day, a small aliquot of DCs (8 × 10^4^) were tested for IL-12 production capability by specific ELISA.

### Vaccination schedule and dosage

It required 7 to 8 weeks to generate sufficient (at least 2 × 10^7^) transfected fibroblasts for each participant. In UPCI 95-033, participants were to receive the first vaccination as soon as this quantity of vaccine cells became available. However, 4 of 6 participants had tumor re-recurrence before the transfected cells proliferated sufficiently, forcing their withdrawal from the study. For both of the participants who received the vaccine in UPCI 95-033, five different formulations were prepared for primary immunization on day 1 by establishing 3 log dilutions of transfected fibroblasts, with the most potent vaccine containing up to 10^7 ^transfected fibroblasts elaborating up to 10^3 ^ng/IL-4/48 hours and admixed with 5 × 10^6 ^irradiated glioma cells. The total number of fibroblasts in each preparation to achieve a given cytokine production level was adjusted by adding non-transfected fibroblasts. Five sites for immunization were oriented vertically and positioned 2 cm apart in the mid-inguinal line over the patient's left thigh. At two weeks after the initial vaccination (on day 15), the 5 immunization sites were punch biopsied and evaluated to determine the dose of IL-4 associated with the most intense mononuclear cell infiltrate. This immunization procedure was repeated on day 15. At this time, 5 immunization sites were placed over the right thigh. Each preparation consisted of as many as 10^7 ^transfected fibroblasts to provide the maximum dose of IL-4 that was found to be free of significant toxicity in primary immunization. Each patient was treated with GCV (Cytovene, Roche Laboratories, Inc., Nutley, NJ; 5 mg/kg × 2/day) from days 8 to 14 and 22 to 28 to ensure elimination of gene-transfected fibroblasts.

In UPCI 99-111, following GTR of the primary tumor, each participant received fractionated external beam radiation therapy (FEBRT; 2 Gy/dose × 30 doses), while gene-transfected autologous fibroblasts were prepared. Following the completion of FEBRT, and as soon as generation of sufficient (at least 2 × 10^7^) transfected fibroblasts was confirmed, DCs for the first vaccine were cultured from peripheral blood-derived monocytes. The first DC vaccine was administered along with IL-4 transfected fibroblasts on day 7 after the peripheral blood draw. On day 7 after the first vaccine, preparation of the second DC vaccine was initiated with fresh peripheral blood-derived monocytes, and thus a course of vaccines was consisted of two intradermal vaccinations with a two week interval (on days 1 and 15), provided that the patient had not suffered a severe local reaction such as abscess or ulceration. Each vaccine formulation was composed of 1 × 10^6 ^lysate-loaded DCs and irradiated (8,000 rads) fibroblasts producing 2 × 10^3 ^ng IL-4/48 h. On the day of each vaccination, before vaccination, the final vaccine formulation was confirmed for the status of sterility, as assessed by absence of gram stain evidence of bacteria, mycoplasma and endotoxin.

### Assessment of safety

Our definitions of toxicity were according to Common Toxicity Criteria Version 3.0 (CTC, v3.0). Regimen-Limiting Toxicity (RLT) was defined as ≥ Grade 2 hypersensitivity reaction and any Grade 3 or greater adverse events with the following exceptions; chills, malaise, fatigue and fever. Because tumor progression was expected in the majority of patients on this protocol, this was not considered a toxicity per se. Similarly, new seizures or changes in seizure frequency were carefully evaluated to determine whether they were related to the study treatment, or to disease progression. If Grade 3 or greater toxicity of any sort, not attributable to disease progression, was detected in >1 of 3 patients or > 2 of 6, then further accrual to the study was to be halted.

### Immunological endpoints

In UPCI 95-033, local immune response was monitored by immunohistochemistry of tissue samples from the vaccine sites [14]. For evaluation of systemic immune response, enzyme linked immuno-spot assay (ELISPOT) assays were performed using PBMC samples collected on days 1 (pre-vaccine), 8, 15 (prior to second vaccine) 22, 30 ± 1 and 60 ± 1. To optimize the sensitivity of the assays, PBMC were re-stimulated *in vitro *for 5 days with low-dose (approx. 50 U/ml) IL-2 and autologous DCs loaded with lysate. Frequencies of IFN-γ producing spots reacting against DC loaded with glioma-lysate were measured as the indicators of type-1 T-cell immune response. For a participant with HLA-A2 haplotype (the second participant in UPCI 95-033), frequencies of IFN-γ producing spots were measured against human glioma-associated, HLA-A2-restricted CTL epitopes IL-13 receptor α2 (IL-13Rα2)_345–353 _[18] and EphA2_883–891 _[19] pulsed onto transporter associated with antigen processing-deficient, HLA-A2^+ ^T2 cells. A participant was considered to have responded, if at each of two consecutive time points, the number of spots was double that at baseline, and there were at least 25 spots/50,000 CD8^+ ^cells.

### Objective clinical response in radiographic analyses

Patients were assessed at one month following their initial vaccination and every two months thereafter for evidence of tumor response using physical examination or radiographic studies. Responses were defined as follows:

A. Complete response – disappearance of all measurable disease for at least 1 month without the development of new lesions.

B. Partial response – 50% or greater decrease in the sum of the radiologically calculated tumor volume of all measurable lesions lasting at least 4 weeks with no increase in the size of existing lesions or appearance of new lesions.

C Minor response – 25–50% decrease in the sum of the radiographically calculated tumor volume of all measurable lesions lasting at least 4 weeks with no increase in the size of existing lesions or appearance of new lesions.

D. Stable disease – no change or < 25% change in the sum of the radiologically calculated tumor volume of all measurable lesions lasting at least 4 weeks with no increase in the size of existing lesions or appearance of new lesions.

E. Progressive disease – any patient manifesting a > 25% increase in the radiologically calculated tumor volume of all measurable lesions or exhibiting the development of new lesions.

## Results

### Treatment of enrolled patients

In UPCI 95-033, only 2 of 6 enrolled participants received scheduled two vaccinations; 4 other participants were withdrawn from the trial because of tumor progression prior to the first vaccination, as it required at least 7 to 8 weeks to generate sufficient numbers of IL-4-transfected vaccine cells (Table 1). Although the trial was initially designed to accrue a total 10 participants, we terminated accrual in this trial after 6 eligible participants because of this major feasibility issue.

**Table 1:** Patient profiles

***95-033***					
**Case #**	**Age**	**Gender**	**Tumor**	**# of previous recurrence**	**Vaccine/Adverse events**

1	63	Male	Lt. Temporal GBM	2	Yes/Grade 2 skin reaction
2	60	Male	Lt. Temporal AA	3	Yes/Grade 1 skin reaction
3	40	Male	Rt. Frontal GBM	3	No due to tumor progression
4	57	Male	Lt. Parieto/Occi GBM	1	No due to tumor progression
5	32	Female	Lt. Parieto/Occi GBM	1	No due to tumor progression
6	53	Female	Rt. Frontal GBM	2	No due to tumor progression
***99-111***					
**Case #**	**Age**	**Gender**	**Tumor**	**TTP (Months)**	**Vaccine/Adverse events**
1	56	Male	Lt. Parietal GBM	4	Yes/Grade 1 headache
2	66	Male	Rt. Frontal GBM	N/A	No due to tumor progression
3	45	Male	Lt. Temporal GBM	10	Yes/None
4	53	Male	Lt. Occipital GBM	6	Yes/None
5	50	Male	Rt. Frontal GBM	6	Yes/None
6	61	Female	Rt. Temporal GBM	4	Yes/None

TTP: Time To Progression

In UPCI 99-111, 5 of 6 enrolled patients received the scheduled two vaccinations (Table 1). The participant who did not receive the vaccines presented with a rapid tumor recurrence immediately after FEBRT. It was decided to terminate accrual in this trial due to: 1) the lack of systemic immunological or clinical responses in 5 treated patients with this regimen; and 2) the dose of DC and number of vaccinations in this study were thought to be suboptimal based on other studies demonstrating encouraging clinical activity of DC-based glioma vaccines [20-22]. This issue is also considered a feasibility issue in the study design.

### Toxicity

No participants experienced regimen-limiting toxicity and there were no deaths on these studies. In UPCI 95-033, grade 2 and 1 skin reactions were seen at the vaccine injection sites in the first and the second participants receiving the vaccines, respectively. In UPCI 99-111, 1 of 5 participants receiving the vaccines reported one transient episode of grade 1 headache that was possibly associated with treatment. There were no other adverse events that were possibly, probably or definitely associated with treatment.

### Production of IL-12 from autologous DCs

**Figure 1:**
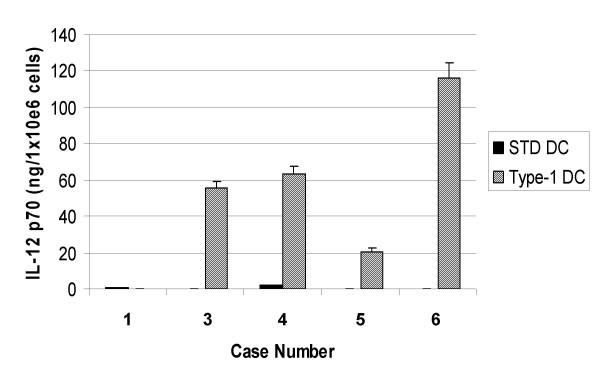
**Type-1 polarized DCs are efficient producers of IL-12 p70**. In UPCI 99-111, on each vaccination day, a small aliquot of DCs (8 × 10^4^) were tested for IL-12 production capability in comparison to DCs that were not stimulated with the type-1 cytokine-mixture (standard DCs). This was done with 24 hr stimulation of DCs (20 × 10^3^per well, duplicates) with CD40L-transfected J558 cells (50 × 10^3^per well). Supernatant was harvested and the production of IL-12 p70 was assayed by specific ELISA. Values indicate averages of duplicate samples. Bars indicate standard errors.

One innovative aspect of UPCI 99-111 trial was that it employed type-1 polarizing DCs [15]. In 5 patients who received vaccines, we evaluated IL-12 p70 production in response to CD40L stimulation using CD40L-transfected J558 cells (Figure 1). In Case 1, only standard DCs were generated because the protocol was amended after case 1 to employ use of type-1, rather than standard DCs. Type-1 DCs were generated for Cases 3 to 6, and produced high levels of IL-12 p70, ranging 20–116 ng/1 × 10^6 ^cells/24 hrs, whereas control standard DCs from the same donors produced only background levels (less than 2 ng/1 × 10^6 ^cells/24 hrs) of IL-12 p70. These results demonstrate that type-1 DCs capable of producing high levels of IL-12 were successfully generated in the current trial.

### Immunological response

**Figure 2:**
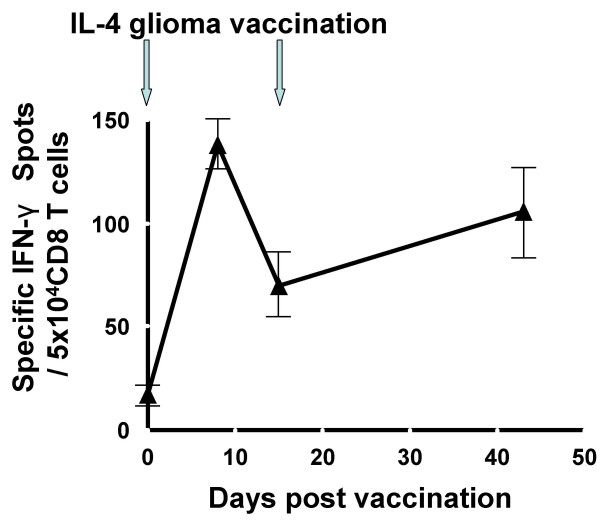
**IL-4 gene transfected glioma cell vaccine elicited an IFN-γ response against EphA2 883–891 epitope**. PBMC samples were obtained on days 1 (pre-vaccine on the day of the first vaccine), 8, 15 (on the day of the second vaccine), and 42, and saved as frozen cells until all these cells were thawed at the same time, cultured in the presence of 20 IU/ml hIL-2 and autologous glioma cells for 5 days, and evaluated for the frequency of IFN-γ-producing cells in response to T2 cells loaded with the HLA-A2-binding EphA2_883–891 _peptide using ELISPOT assay. Each well contained 5 × 10^4 ^CD8^+ ^cells and each group was evaluated in triplicate. Specific IFN-γ spots were calculated by subtracting the average of control spots (triplicate variation within a group was less than 10% in non-peptide-loaded T2 cell groups) from the total numbers of spots in peptide-loaded groups. Values indicate averages of triplicate samples for each time point, and bars indicate standard deviations. The number of spots in each post-vaccine time point was at least three times the standard-deviation of the pre-vaccine value.

In UPCI 95-033, two participants who received vaccinations against their recurrent GBM demonstrated local (the first patient) or systemic (the second patient) immunological responses. Biopsies from the vaccine sites of the first patient displayed IL-4 dose-dependent infiltration of CD4^+ ^and CD8^+ ^T cells [23]. Although ELISPOT assays did not demonstrate a systemic immune response against autologous glioma cells in this patient, the second vaccine recipient in this trial was HLA-A2-positive, allowing us to evaluate the induction of systemic T-cell responses against the HLA-A2-restricted GAA epitopes EphA2_883–891 _[19] and IL-13Rα2_345–353 _[18] using ELISPOT assays. In this patient, vaccinations using autologous glioma as the antigen source induced a specific IFN-γ response against EphA2_883–891 _that was sustained for at least 6 weeks post-vaccination (Figure 2), although a specific response against IL-13Rα2_345–353_or autologous bulk glioma cells was not detectable in the same assays. No PBMC samples were collected at any later time points, and thus we were unable to monitor the persistence of such responses for longer periods of time. Immunohistochemical analysis of the vaccine site biopsy sample, which was performed in the first patient [23], was not performed for the second patient due to suboptimal sample quality.

In 99-111, we were unable to detect increased IFN-γ responses in any of 5 patients who received the scheduled two vaccines.

### Clinical response

**Figure 3:**
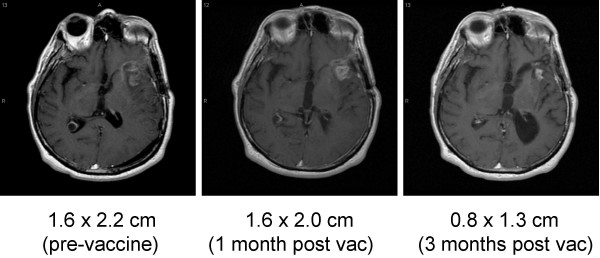
**Regression of recurrent glioblastoma following vaccination with autologous glioma cells admixed with IL-4-transfected fibroblasts**. Axial gadolinium-enhanced T1-weighted MR images obtained from the 2^nd ^participant in UPCI 95-033. Enhanced lesions were measured independently by a neuroradiologist.

In UPCI 95-033, despite the fact that only of two patients completed the protocol-defined sequence of vaccinations, these two patients, both of whom had recurrent GBM, demonstrated radiological improvement during the 4 months after vaccination [[23] and (Figure 3)]. However, both patients eventually succumbed to tumor recurrence, which started at 6 months following the first vaccination.

In UPCI 99-111, 5 patients received two vaccinations, and time to progression was 4, 10, 6, 6 and 4 months, respectively, after resection of newly diagnosed GBM (Table 1).

## Discussion

We described our results from two gene therapy trials in patients with recurrent or newly diagnosed malignant glioma, UPCI 95-033 and 99-111, respectively. We identified common challenges in the administration of these protocols. Both protocols utilized participants' autologous fibroblasts that had to be stably transfected with the TFG-hIL4-Neo-TK retroviral vector, and expanded to sufficient numbers. A retroviral vector was chosen because of stable expression and positive efficacy data in preclinical models [6,7]. However, these processes required at least 7 to 8 weeks, which posed a major feasibility concern. Especially in the UPCI 95-033 trial, 4 of 6 participants were withdrawn from the study due to tumor re-recurrence before they could receive the first vaccine. In addition, the first patient who received the vaccines had a rapid recurrence after the surgery for tumor debulking and vaccine cell harvesting, and underwent another tumor resection before beginning vaccination [23]. Indeed, in the literature [24], the median time to further tumor progression for patients with recurrent malignant glioma, even with therapy, is only 8 weeks. We performed skin harvesting during the craniotomy of recurred tumor for UPCI 95-033. As craniotomy and debulking surgery are usually performed as soon as these interventions are clinically indicated, there are no practical ways to perform skin harvesting and transfection earlier. In UPCI 99-111, this issue was not as significant as in UPCI 95-033 as only one of 6 participants was withdrawn due to tumor recurrence before the first vaccine. In this trial, standard FEBRT, which typically requires 6 weeks (2 Gy/dose × 30 doses), was used following surgical resection of primary tumors, and this may have temporarily suppressed tumor growth and provided an adequate time for vaccine generation and post-irradiation delivery.

The primary endpoint of these studies was evaluation of safety, and we did not observe any regimen-limiting toxicity. Because autologous whole glioma cells were used as the source of the vaccines, there was a theoretical concern for inducing autoimmune encephalitis. Although the lack of autoimmune events in our two trials is reassuring in this regard, it does not indicate the ultimate safety of such approaches, and continued vigilance for such toxicity is warranted, particularly because only 7 evaluable participants were accrued. It has been demonstrated that the clinical benefit of immunotherapy in other types of cancers is often associated with induction of autoimmunity [25]. On the other hand, patients with advanced cancers and compromised immune systems may not be ideal candidates for assessing either the toxicity or efficacy of therapeutic cancer vaccines [26]. Further evaluation of these important issues is warranted in larger numbers of glioma patients receiving current, more intensive, vaccine regimens.

With regard to induction of immune response, two participants in UPCI 95-033 who received vaccines composed of IL-4-transfected fibroblasts and irradiated autologous glioma cells demonstrated either local (i.e. immune cell infiltrate at the vaccine site) or systemic (i.e. IFN-γ ELISPOT response against EphA2-derived epitope) immune responses. These observations are consistent with previous clinical trials in our institute that local production of IL-4 by gene-transfected fibroblasts at the vaccine site caused local endothelial activation and recruitment of immune effectors [27,28]. On the other hand, no evidence of systemic immune response was observed in five participants in UPCI 99-111 who received IL-4-transfected fibroblasts admixed with DCs loaded with tumor-lysate. These results were somewhat surprising because we had expected that the vaccine formulation using type-1 DCs would be more potent than the formulation in UPCI 95-033. In UPCI 99-111, each participant received only two intradermal injections of 1 × 10^6 ^lysate-loaded type-1 DCs. The dose of DC and number of vaccinations in this study were probably suboptimal based on other studies demonstrating encouraging clinical activity of DC-based glioma vaccines [20-22]. In these studies, higher DC numbers and repeated vaccinations (1 × 10^7^/injection × 3 or more vaccinnations) were employed.

In addition to the intensity of vaccine regimens, careful consideration is required for optimal administration routes of DC vaccines. In a randomized study comparing direct intra-lymph-nodal, intravenous and intradermal administration of DC vaccines in patients with metastatic melanomas [29], the intra-lymph-nodal route was well-tolerated for up to 5 × 10^7 ^DCs/injection, and induced significantly higher levels of specific CD8^+ ^T cells based on cytokine secretion, when compared with other routes. The feasibility, safety and efficacy of intra-lymph-nodal administration of DC-based vaccines has also been demonstrated using the activation of antigen-specific CD4^+ ^T cell responses as an endpoint [30]. Therefore, in our currently ongoing type-1 DC-based trials (UPCI 04-136 and 05-115), we selected ultrasound-guided intra-lymph-nodal injections of type-1 DCs.

Novel type-1 DCs were employed in UPCI 99-111, and these DCs generated from each participant demonstrated enhanced IL-12 p70 producing capability *in vitro *compared to standard DCs from the same donors. These results indicate that we have established standard operation procedures for GMP-grade type-1 DCs for our ongoing and future type-1 DC-based trials. In addition, further improvement of the DC culture protocol has lead to development of a serum-free culture protocol for type-1 DCs [15]. Type-1 DCs are expected to induce type-1 immune effector cells, including T-helper (Th)1 and type-1 cytotoxic T-lymphocytes (Tc1). Our recent studies in murine brain tumor models have demonstrated that type-1 T-cell response is particularly favorable for anti-brain tumor immunotherapy owing to high level surface expression on Tc1 cells of a type-1 chemokine receptor CXCR3 [31] and an integrin, very late antigen-4 [32], both of which mediate critical roles in efficient trafficking of anti-tumor T-cells to the brain tumor site. Therefore, our ongoing and future development of glioma vaccine-based immunotherapy is directed towards induction of effective and safe type-1 immunity in patients suffering from brain tumors.

Although IL-4 is typically described as an inducer of a type-2 immune response [33], local delivery of IL-4 at an immunization site induced IL-12 production by antigen-presenting cells, and Th1-type responses in our pre-clinical tumor vaccine model [34] as well as in an infectious disease (Leishmania major) model [35]. Interestingly, in this Leishmania major model [35], systemic or prolonged recombinant (r)IL-4 delivery rather induced Th2-response, suggesting bi-functional roles of IL-4 depending upon target cell types (i.e. antigen-presenting cells vs. T-cells). Indeed, *in vitro *exposure of DCs to IL-4 promotes IL-12 secretion from DCs [10-12].

We also evaluated preliminary therapeutic activity of these regimens. In two participants in UPCI 95-033 who received scheduled vaccines, temporary stabilization of disease and radiological improvement was observed for 4 to 6 months. However, the clinical course of the first patient after vaccination was complex due to transiently increased gadolinium enhancement on MRI scans at 2 to 3 months after vaccination [23]. This transient worsening may not have been true tumor progression, but pseudo-tumor progression representing inflammatory response at the tumor site. Biopsy of the tumor would have been the ultimate modality to distinguish these two scenarios. As we currently employ more intensified vaccine regimens (discussed in the next paragraph), the distinction between true- and pseudo-tumor progression is becoming a more critical issue. Biopsy may not always be clinically justifiable in patients with recurrent glioma. The utility of MR spectroscopy and metabolic positron emission tomography (PET) imaging for differentiating tumor progression from immune-mediated inflammatory treatment effect remains to be established. In 99-111, the median time to progression after surgical resection in 5 participants who received scheduled vaccines was 6 months (ranging 4 to 10 months); and this is not superior to the current standard regimen with FEBRT and temozolomide (6.9 months) or FEBRT alone (5.0 months) [36]. The low intensity of the vaccine regimen in the UPCI 99-111 study, with only 1 × 10^6 ^DC/injection × two injections, may have limited the manifestation of any therapeutic immune response. Future studies will need to determine whether intensification of the vaccine approach can enhance therapeutic efficacy without a counterbalancing induction of detrimental autoimmunity.

Although our analyses of IL-4 tumor vaccine models have led us to understand the critical roles of type-1 immunity for brain tumor immunotherapy, effective type-1 immunity may also be achieved by clinically more feasible modalities than IL-4 transfected fibroblasts. Indeed, our recent preclinical study has demonstrated that a Toll-like receptor-3 ligand poly-ICLC, which has been extensively evaluated for safety in patients with glioma [37], enhances type-1 anti-glioma immunity when combined with peripheral vaccine regimens [38]. We have also isolated and characterized HLA-A2-restricted CD8^+ ^CTL epitopes derived from human GAAs, such as IL-13Rα2 [18] and EphA2 [19] to develop vaccines using "off the shelf" synthetic peptides for these epitopes. Based on these studies, we recently implemented vaccine trials evaluating safety and immunological activity of type-1 inducing DCs loaded with synthetic peptides encoding these GAA-derived CTL epitopes in HLA-A2^+ ^patients with recurrent glioma (UPCI 04-136 and 05-115). In these trials, to date, 7 of 8 participants successfully received 4 scheduled vaccinations with 2-week intervals without tumor recurrence, suggesting improved feasibility of these novel vaccine approaches.

Our ongoing progress in basic understanding in brain tumor immunology will allow us to develop more efficient and feasible immunotherapy strategies for patients suffering from these intractable diseases.

## Competing interests

The author(s) declare that they have no competing interests.

## Authors' contributions

HO and IFP designed these trials, and played central roles in patient-accrual and regulatory oversights. FSL, AT, JLM, SCS and HO played central roles in clinical management of participants. KAW, LDL, DSK and GKB performed neurosurgical procedures to obtain tumor tissues. RLH reviewed tumor tissues for neuropathological diagnosis. PK developed the DC culture protocol for UPCI 99-111. QC characterized transfected fibroblasts for absence of replication competitive retroviruses. HDE harvested skin grafts for fibroblast culture. LHB and TLW supervised and conducted analyses of lymphocyte-subsets in PBMCs. DMP provided statistical considerations during the design of these trials, although studies were terminated before scheduled numbers of patients were accrued for analyses.
